# Enhanced Osteogenic Differentiation of Human Bone Marrow-Derived Mesenchymal Stem Cells by a Hybrid Hydroxylapatite/Collagen Scaffold

**DOI:** 10.3389/fcell.2020.610570

**Published:** 2021-01-11

**Authors:** Elisa Mazzoni, Chiara Mazziotta, Maria Rosa Iaquinta, Carmen Lanzillotti, Francesca Fortini, Antonio D’Agostino, Lorenzo Trevisiol, Riccardo Nocini, Giovanni Barbanti-Brodano, Andrea Mescola, Andrea Alessandrini, Mauro Tognon, Fernanda Martini

**Affiliations:** ^1^Department of Medical Sciences, School of Medicine, University of Ferrara, Ferrara, Italy; ^2^Maria Cecilia Hospital, GVM Care & Research, Cotignola, Italy; ^3^Department of Surgical Odonto-Stomatological Sciences, University of Verona, Verona, Italy; ^4^Department of Oncologic and Degenerative Spine Surgery, IRCCS Istituto Ortopedico Rizzoli, Bologna, Italy; ^5^CNR-Nanoscience Institute-S3, Modena, Italy; ^6^Department of Physics, Informatics and Mathematics, University of Modena and Reggio Emilia, Modena, Italy

**Keywords:** scaffold, bone, expression, gene, osteogenesis

## Abstract

Human bone marrow-derived mesenchymal stem cells (hBMSCs) and their derivative enhanced green fluorescent protein (eGFP)-hBMSCs were employed to evaluate an innovative hybrid scaffold composed of granular hydroxylapatite and collagen hemostat (Coll/HA). The cellular morphology/cytoskeleton organization and cell viability were investigated by immunohistochemistry (IHC) and AlamarBlue metabolic assay, respectively. The expression of osteopontin and osteocalcin proteins was analyzed by IHC and ELISA, whereas osteogenic genes were investigated by quantitative PCR (Q-PCR). Cell morphology of eGFP-hBMSCs was indistinguishable from that of parental hBMSCs. The cytoskeleton architecture of hBMSCs grown on the scaffold appeared to be well organized, whereas its integrity remained uninfluenced by the scaffold during the time course. Metabolic activity measured in hBMSCs grown on a biomaterial was increased during the experiments, up to day 21 (*p* < 0.05). The biomaterial induced the matrix mineralization in hBMSCs. The scaffold favored the expression of osteogenic proteins, such as osteocalcin and osteopontin. In hBMSC cultures, the scaffold induced up-regulation in specific genes that are involved in ossification process (BMP2/3, SPP1, SMAD3, and SP7), whereas they showed an up-regulation of MMP9 and MMP10, which play a central role during the skeletal development. hBMSCs were induced to chondrogenic differentiation through up-regulation of *COL2A1* gene. Our experiments suggest that the innovative scaffold tested herein provides a good microenvironment for hBMSC adhesion, viability, and osteoinduction. hBMSCs are an excellent *in vitro* cellular model to assay scaffolds, which can be employed for bone repair and bone tissue engineering.

## Introduction

For many years, hundreds of laboratories have performed studies aimed at unraveling the biological characteristics of mesenchymal stem cells (MSCs) and probing the modalities of their potential contribution to cartilage and bone repair (Barry, 2019). In cell therapy and tissue engineering, it is well established that human bone marrow-derived MSCs (hBMSCs) play an important role in tissue healing/regeneration due to their self-renewal, migration, and pluripotency characteristics. For these reasons, hBMSCs are the most frequently used stem cells for tissue repair (Liu et al., 2017; Fu et al., 2019). However, hBMSCs need to migrate from the bone marrow to injured tissues during the healing process, through peripheral circulation as a prerequisite (Fu et al., 2019). In recent years, the crucial repairing action of hBMSCs in damaged and diseased tissues was reported by several experimental studies (Fu et al., 2019). hBMSCs are able to migrate into damaged tissues and perform wound healing through two key mechanisms, that is, paracrine and direct differentiation. Indeed, the migration of hBMSCs is regulated by mechanical and chemical factors in this trafficking process (Imura et al., 2019).

It has been demonstrated that during this repairing process, recruited hBMSCs secrete a combination of chemical factors, such as chemokines, cytokines, and growth factors, which act in a paracrine manner to promote tissue repair (Fu et al., 2019). Once hBMSCs arrive into the injured site, they proliferate and osteogenically differentiate. Osteogenic differentiation of hBMSCs is a complex process regulated by multiple factors. Among these, the characteristics of the scaffold (i.e., composition and structure) can regulate cellular fate. Due to age-related illnesses, accidents, risky sports, and tumor resections, the need for bone grafts is high (Mazzoni et al., 2015; Ottensmeyer et al., 2018). There is an urgent clinical need for an alternative therapeutic strategies aimed at bone healing for the ever-increasing number of bone grafting procedures performed annually (Sheehy et al., 2019). Unfortunately, both autograft and allograft have intrinsic disadvantages, including the volume of collectable autologous bone and patient morbidity, or immunogenic rejection and risk of disease transmission. For these reasons, the use of scaffolds is on the rise in translational medicine for bone repair/regrowth. Many efforts have been carried out to develop innovative laboratory-produced tissue replacements. However, current approaches to bone tissue engineering usually lack sufficient functionality respect to native bone matrix (Sheehy et al., 2019; Weiss-Bilka et al., 2019).

An ideal scaffold for bone fracture treatment and regeneration must combine osteoconduction, osteoinduction, and osteogenesis proprieties. The hydroxylapatite (HA)-derived scaffold, commonly produced via several synthetic routes, overtime, have been found to be good material for significant parameters, including bioactivity, biocompatibility, and osteoconductivity both *in vitro* and *in vivo.* In order to mimic the 3D architecture of trabecular bone, HA is an excellent candidate as a substitute to natural bone (Ramesh et al., 2018). In contrast to the brittle nature of HA, it has been combined with several polymers in the form of biocomposite implants in order to primarily improve its mechanical properties (Ramesh et al., 2018). The concept of developing a collagen (Col)–HA biocomposite is justified by the fact that it constitutes bone microarchitecture (Ramesh et al., 2018). Type I Col is the main molecule of interest when it comes to bone regeneration, as it is abundantly found in bone (Sherman et al., 2015). Thus, biocomposite scaffolds composed of Col reinforced with HA are an attractive choice for bone tissue engineering since their composition mimics bone (Weiss-Bilka et al., 2019). Hybrid HA/Col scaffold, composed of Granular Pro Osteon^®^ 200 coralline HA and Avitene^TM^ Microfibrillar Collagen Hemostat (Coll/Pro Osteon^®^ 200), is used during malarplasty in maxilla-facial patients, producing a successful outcome and promoting bone formation (D’Agostino et al., 2016; Mazzoni et al., 2020). Col–HA biocomposites have shown enhanced cytocompatibility compared with pure Col scaffolds. *In vitro* studies have been reported to have improved attachment and proliferation of different cell lines, including MG63 osteosarcoma cells, MC3T3 osteoblast precursors, human osteoblasts, and L-929 fibroblasts on this scaffold (Ramesh et al., 2018). In our *in vitro* model of human adipose MSCs (hASCs), Coll/Pro Osteon^®^ 200 can induce the expression of significant genes involved in skeletal development (Mazzoni et al., 2017, 2020).

At present, several types of MSCs have been identified as a source of osteoblast progenitors; in this context, it is necessary to analyze different types of MSCs using *in vitro* approaches in order to evaluate the potential of MSCs from various origins and select the best source for cell-based therapy. Being armed with a considerable number of MSC features and the molecular mechanisms modulating osteoblast differentiation of MSCs, alteration in MSCs will make MSC-based cell therapy harmless and more operative for clinical use in the future. However, there are still controversial hypotheses regarding which MSC types can be used in regenerative medicine.

Herein, we evaluate hBMSC morphology, viability, and differentiation analyzing expressed genes in hBMSCs grown on the biocomposite scaffold Coll/Pro Osteon^®^ 200, which was not employed yet in oral maxillofacial surgery.

## Materials and Methods

### In vitro Biocompatibility Assays Carried Out on a Hybrid Hydroxylapatite/Collagen Scaffold Using Human Bone Marrow-Derived Mesenchymal Stem Cells

Human bone marrow-derived mesenchymal stem cells and their derivative enhanced green fluorescent protein (eGFP)-hBMSCs were employed to evaluate an innovative hybrid scaffold composed of granular HA (Pro Osteon^®^ 200, Interpore Cross Irvine, CA, United States) and Avitene Col hemostat (Bard Warwick, RI, United States) (Coll/HA).

### Cell Culture Preparation and Fluorescence-Activated Cell Sorting Characterization

Iliac crest bone marrow aspirates (10 ml) were obtained from orthopedic patients who underwent bone marrow harvesting under general anesthesia. Specimens were obtained according to the tenets of the Declaration of Helsinki and the Ethical Committee of the Orthopedic Institute “Rizzoli,” Bologna, Italy. All donors provided informed written consent for the biopsy. A mononuclear fraction was isolated by Ficoll-mediated (Histopaque, 10771, Sigma Company, Milan, Italy) discontinuous density gradient centrifugation and polystyrene adherence capacity, as reported (Manfrini et al., 2013). Analyses were performed in triplicate employing three different samples of iliac crest bone marrow aspirates from three patients. hBMSCs were cultured in α-minimum essential medium (α-MEM) (Lonza, Milan, Italy) supplemented with 20% fetal bovine serum (FBS) and 2% antibiotics (Pen/Strep 10,000 U/ml) at the density of 5,000 cells/cm^2^, in a T75 flask (Falcon BD, Franklin Lakes, NJ, United States) at 37°C with 5% CO_2_ in a humidified atmosphere. After isolation, hBMSCs were characterized by flow cytometry analysis (FCA) using several positive (CD29, CD73, and CD90) and negative (CD14 and CD45) MSC surface markers, as previously reported (Manfrini et al., 2013). At the second passage, hBMSCs were randomly assigned to three experimental groups: (i) hBMSCs grown on the biomaterial Coll/Pro Osteon^®^ 200; (ii) hBMSCs grown in osteogenic condition (OC); and (iii) hBMSCs grown in monolayer in 24-well tissue culture polystyrene (TCPS) plates, employed as control. In the biomaterial group, the scaffold was placed separately in 24-well plates (Ø = 10 mm) to cover the surface area. hBMSC cultures were then filled with 20 μl of cell suspension containing 10^4^ cells for each sample and incubated for 2 h (Manfrini et al., 2013). Cell suspension was subjected to shaking every 15 min in order to maximize cell-scaffold interaction. In the OC group, hBMSCs were cultured in hBMSCs differentiation Bullekit^TM^ osteogenic medium (Lonza, Milan, Italy), which contains osteogenic basal medium (Lonza, Milan, Italy) and osteogenic SigleQuotes^TM^ (dexamethasone, ascorbate, mesenchymal cell growth supplement, L-glutamine, and β-glycerophosphate) (Lonza, Milan, Italy) (Mazzoni et al., 2020). Cultures were maintained at 37°C, 5% CO_2_ up to 3 weeks, whereas the medium was replaced every 2 days.

### Scaffold Preparation and Scanning Electron Microscopy Characterization

Porous HA-derived scaffolding employed herein is composed of Granular Pro Osteon^®^ 200 (Interpore Cross Irvine, CA, United States) and Avitene^TM^ Microfibrillar Collagen Hemostat (Bard Warwick, RI, United States) (Coll) (D’Agostino et al., 2016; Mazzoni et al., 2017, 2020). Granular Pro Osteon^®^ 200 is a coralline HA, which is very similar in its makeup to human bone mineral composition and form. The manufacturing scaffold used *in vitro* evaluations was described before in detail (D’Agostino et al., 2016; Mazzoni et al., 2017, 2020). The innovative scaffold, named Coll/Pro Osteon 200, is composed of two commercially available materials: (i) Avitene^TM^ Microfibrillar Collagen Hemostat from Bard Warwick, RI, United States, and (ii) Pro Osteon^®^ 200, from Interpore Cross Irvine, CA, United States. However, the hybrid scaffold is prepared by the operator at time of the surgery, just mixing in the right proportion the two products, i.e., granules of Pro Osteon^®^ 200 (5 ml) are mixed with 1 g of Avitene^TM^ Microfibrillar Collagen Hemostat and 5 ml of sterile water to make a malleable scaffold. The mixture is prepared to obtain several small disks (Ø, 1 cm; height, 0.2 cm). These blocks of biomaterial (*n* = 20–25) are left overnight to dry under UV light. The biomaterial was analyzed by scanning electron microscope (SEM) (Cambridge United Kingdom, model Stereoscan S-360) (Mazzoni et al., 2017). Samples were washed with saline and fixed for 1 h by 2.5% glutaraldehyde and additional 4 h with a 1% osmium solution in phosphate buffer. The biomaterial was coated with colloidal gold and SEM analyzed (D’Agostino et al., 2016; Mazzoni et al., 2017, 2020).

### Atomic Force Microscopy Measurements

Atomic force microscopy (AFM) analysis was performed with a BioScope I microscope equipped with a Nanoscope IIIA controller (Veeco Metrology, Plainview, NY, United States). A custom temperature control system based on the use of Peltier cells was exploited to keep the sample temperature at 37°C; briefly, Peltier cells were connected to a custom-developed control unit exploiting the proportional integral derivative (PID) feedback system of an Arduino microcontroller. One of the two surfaces of the Peltier cells was in contact with the petri dish via a metal support, while the opposite side of the cells was in contact with a circulating water thermal bath whose temperature was controlled by a temperature control unit (Lauda-Brinkmann, Delran, NJ, United States). The sample’s temperature was checked by a digital thermometer Fluke 16 (Fluke, Brugherio, Italy) equipped with a small K-thermocouple probe (Thermocoax, Heidelberg, Germany). Mechanical characterization of cells was performed by force spectroscopy measurements using triangular silicon nitride cantilevers (Bruker DNP-S) with nominal spring constants of 0.06 N/m. In particular, AFM was used in the force-volume mode that allows to obtain two-dimensional maps of the sample mechanical properties in which each pixel corresponds to a force-indentation curve; at the same time also, height information of the sample was stored. Force-volume maps were analyzed using the Sneddon model fitting procedure of the Nanoscope analysis software (version 1.8) to obtain the Young modulus for each force curve.

### Cell Viability, Cytoskeleton Architecture, and Metabolic Activity

Human bone marrow-derived mesenchymal stem cells (10^4^ cells) were seeded onto the Coll/Pro Osteon^®^ 200 scaffold in order to evaluate the influence of the scaffold on viability and cytoskeleton organization. hBMSCs from the three experimental groups, i.e., biomaterial, OC and TCPS, were assayed at different time points (days 14 and 21). *Cell viability:* To facilitate the observation of hBMSC cultures grown on the biomaterial, cells were transfected with an adenovirus vector expressing the eGFP. Recombinant Ad-GFP was prepared as described previously (Kim et al., 2008; Mazzoni et al., 2020). After 48 h, the efficiency of the adenovirus infection was evaluated by measuring the emitted fluorescence using a fluorescence microscope. *Cytoskeleton architecture:* Cytoskeletal actin filaments of hASCs-eGFP were stained with tetramethyl-rhodamine-iso-thio-cyanate (TRITC) conjugated phalloidin (Sigma, Milan, Italy) at day 14, as previous described (Manfrini et al., 2013; Mazzoni et al., 2017, 2020). *Cell viability assay:* The viability rate of hBMSC cells grown with the biomaterial was determined using the AlamarBlue^TM^ assay (Invitrogen, Milan, Italy). The assay was carried out to evaluate the viability of cells attached and grown on the biomaterial and control (TCPS) at days 14 and 21 (Manfrini et al., 2013; Mazzoni et al., 2017). Briefly, cells were incubated with a solution of 10% AlamarBlue in medium for 3.5 h at 37°C. For the AlamarBlue assay, a calibration curve with scalar concentrations of hBMSCs (10^3^–10^5^ cells) was generated. Afterward, the optical density of the supernatants was measured at 570 and 620 nm using the spectrophotometer (Thermo Electron Corporation, model Multiskan EX, Helsinki, Finland). Biocompatible analysis was carried out in triplicate for each biological sample under investigation.

### Osteopontin and Osteocalcin Expression

Immunofluorescence staining was carried out at day 21 of culture to detect osteopontin (OPN) and osteocalcin (OCN) proteins. The expression of these proteins was investigated in hBMSC grown on the biomaterial (B), in OCs and in the control group (TCPS). Experiments were carried out in triplicate for each biological sample analyzed. hBMSCs grown on the Coll/Pro Osteon 200^®^ scaffold for 14 days were washed twice with PBS and fixed for 20 min with 4% paraformaldehyde (PFA) (Sigma, Milan, Italy) in order to identify OPN and OCN protein expression. After three PBS washes, cells were treated for 10 min with 0.1% Triton X-100, washed twice with PBS 1×, and incubated for 1 h at room temperature (RT) with a rabbit polyclonal anti-OPN (Thermo Fisher Scientific, Milan, Italy) or anti-OCN (Thermo Fisher Scientific, Milan, Italy) antibody (both at 1:100 dilution) in PBS for 16 h at 4°C. After two PBS washes, cells were incubated for 1 h at RT with Donkey anti-Rabbit IgG (H+L) Highly Cross-Adsorbed Secondary Antibody, Alexa Fluor^®^ 488 conjugate (Thermo Fisher Scientific, Milan, Italy). Fluorescent images were taken using a TE2000E fluorescence microscope (Nikon Instruments Spa, Sesto Fiorentino, Italy). Digital images were captured using ACT-1 and ACT-2 software for DXM1200F digital cameras (Nikon Instruments Spa, Sesto Fiorentino, Italy). Nuclei were stained with 0.5 mg/ml of 4,6′-diamino-2-phenylindole (DAPI) (Mazzoni et al., 2017, 2020). ELISA was performed at days 14 and 21 in order to quantify the OCN protein. OCN was extracted with Cell Extraction Buffer (Thermo Fisher Scientific, Milan Italy) added to 1 mM of phenylmethylsulfonyl fluoride (PMSF), in the presence of a protease inhibitor cocktail (Mazzoni et al., 2020). The concentration of total proteins was determinate using the bicinchoninic acid (BCA) assay according to the manufacturer’s instructions (Mazzoni et al., 2020). The osteocalcin protein was quantified by the Human Osteocalcin Instant ELISA (Thermo Fisher Scientific, Milan, Italy) according to the manufacturer’s instructions (Mazzoni et al., 2020).

### Alizarin Red Staining

Alizarin red (AR) (Sigma, Milan, Italy) was used to analyze the matrix mineralization, as described (Mazzoni et al., 2017, 2020). AR staining was carried out at days 14 and 21. The mineralized substrates were quantified using 20% methanol and 10% acetic acid in a water solution (Sigma-Aldrich, Milan, Italy). Quantification of matrix mineralization was carried out in triplicate for each biological sample analyzed. Images were taken using a standard light microscope (Nikon Eclipse TE 2000-E microscope, Nikon Instruments Spa, Sesto Fiorentino, Italy) equipped with a digital camera (DXM 1200F; Nikon Instruments Spa, Sesto Fiorentino, Italy). In order to quantify the matrix mineralization, the solution was transferred into cuvettes, whereas the quantity of AR dissolved was read spectrophotometrically (Thermo Electron Corp., model Multiskan EX, Vantaa, Finland) at a wavelength (λ) of 450 nm (Mazzoni et al., 2017, 2020).

### Osteogenesis RT^2^ Profiler PCR Array

Osteogenesis PCR array was performed, in triplicate for each biological sample, in hBMSC cultures grown on the biomaterial in order to identify genes from the osteogenic pathway activated by the scaffold. Specifically, total RNA was isolated using the RNeasy Plus Micro Kit (Qiagen, Milan, Italy) (Mazzoni et al., 2020) according to the manufacturer’s instructions from cells grown on (i) Coll/Pro Osteon^®^ 200 scaffolding and (ii) TCPS (control group) (Manfrini et al., 2013). RNA was quantified using a NanoDrop spectrophotometer (ND-1000; NanoDrop Technologies, Wilmington, DE, United States) (Mazzoni et al., 2017, 2020). The Human Osteogenesis RT^2^ Profiler PCR Array (Qiagen, Milan, Italy) was used as described (Mazzoni et al., 2020). Specific primer sets employed in real-time PCRs were used to analyze the expression of 84 genes involved in different pathways, such as osteogenic differentiation, cartilage condensation, ossification, bone metabolism, bone mineralization, binding to Ca^2+^ and homeostasis, extracellular matrix (ECM) protease inhibitors, adhesion molecules, cell-to-cell adhesion, ECM adhesion molecules, and growth factors. All reactions were performed in triplicate. For data analysis, the fold change (FC) of each gene expression was calculated using the 2^–ΔΔ*Ct*^ method, whereas housekeeping genes, employed as controls, were used to normalize results and Log_2_ FC; < 1 or >1 was considered significant (Mazzoni et al., 2017).

### Statistical Analysis

Data are expressed as a mean of standard deviation. Statistical analyses of experiments, which were performed in triplicate, were carried out using Prism6 software (GraphPad 6.0, San Diego, CA, United States). Data obtained from AlamarBlue assay were analyzed with the *t*-test. To analyze the osteocalcin protein and matrix mineralization, we used one-way analysis of variance (ANOVA) with Dunnett post-test analysis (Mazzoni et al., 2014, 2020). A value of *p*-value < 0.05 was considered significant.

## Results

### Human Bone Marrow-Derived Mesenchymal Stem Cell Flow Cytometry Markers of Stem Cells

**FIGURE 1:**
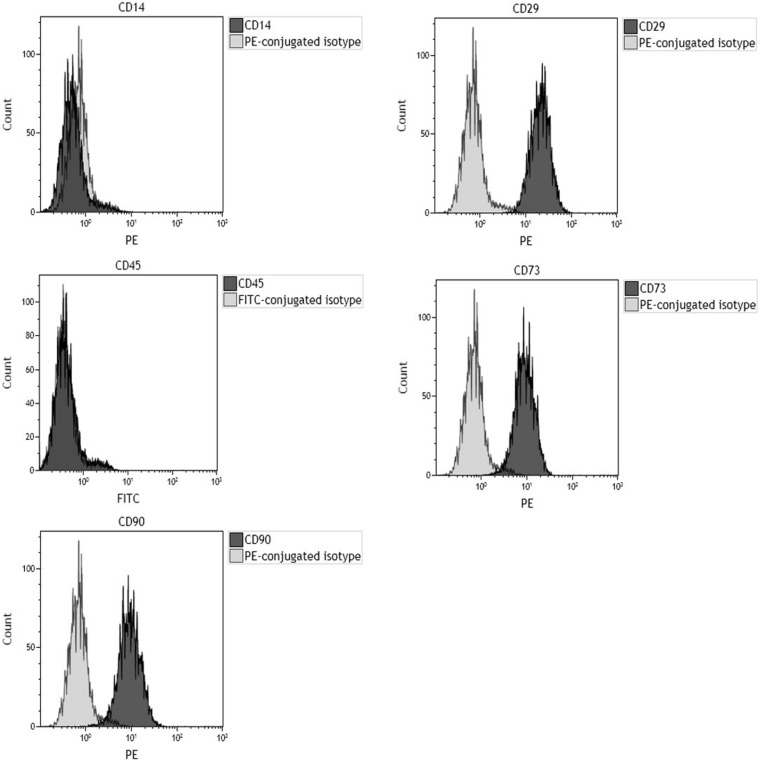
Flow cytometric characterization of cell-surface antigen profile of human bone marrow-derived mesenchymal stem cells (hBMSCs) obtained from bone marrow of orthopedic patients. Plots are representative of three distinct analyses, employing hBMSCs derived from a single patient. In each graph, the dark gray histogram plot represents sample stained with the indicated antibody, whereas the light gray histogram plot represents isotype control. CD29, CD73, and CD90 represent hMSC-positive surface markers, while CD14 and CD45 are hMSC-negative surface markers. Phenotypic analysis confirmed that cells tested positive for hMSC markers (CD29, CD73, and CD90) and negative for hematopoietic (CD45) and macrophage (CD14) markers.

Flow cytometry analysis for specific surface antigens was carried out to evaluate hBMSC markers. As expected, surface antigen profiles matched markers indicated from the International Society for Cell & Gene Therapy guidelines. Purity level of hBMSCs in the samples was 99% toward CD29, CD73, and CD90 expression. hBMSCs resulted negative for hematopoietic (CD 45) and macrophage (CD14) markers in phenotypic analysis (Figure 1).

### Scanning Electron Microscopy Characterization of Coll/Pro Osteon^®^ 200 Scaffolding

**FIGURE 2:**
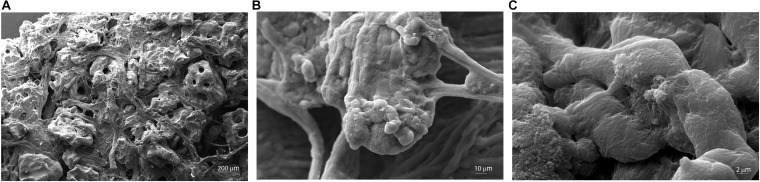
Scanning electron microscopy (SEM) analysis of the Coll/Pro Osteon^®^ 200. **(A)** Coll/Pro Osteon^®^ 200 shows the porous structure with several pores within a range of 190–230 μm, magnification 77×. **(B,C)** Organization and structure of collagen fibrils bovine from Avitene^TM^ Microfibrillar Collagen Hemostat (Bard Warwick, RI, United States) visualized on Coll/Pro Osteon^®^ 200. Collagen fibrils randomly organized are present as fibril interweaving insert maintained the porous structure (2.01KX; 10.73KX).

The microstructure and morphology of the scaffold (Coll/Pro Osteon^®^ 200) were analyzed through SEM. Granular HA (Pro Osteon^®^ 200) mixed with Col fibers Col (Avitene^TM^ Microfibrillar Collagen Hemostat) generates a highly fibrous structure (Figure 2). Some evidence of splaying was noted (e.g., fibers twisted around one another and a mixture of fibers branching). Figure 2A shows the porous scaffold structure with the regular pore sizes in the range of 190–230 μm. Fibril interweaving of Col fibrillary in Avitene^TM^ Microfibrillar Collagen Hemostat Fluor organization inserted on the scaffold was observed at higher magnification (2.01–10.73KX) (Figures 2B,C). The scaffold exhibited Col fiber microstructural features, such as fusing/bifurcating fibrils (Figures 2B,C) rotating at a porous structure of HA.

### Scaffold Biocompatibility Analysis With Human Bone Marrow-Derived Mesenchymal Stem Cells

**FIGURE 3:**
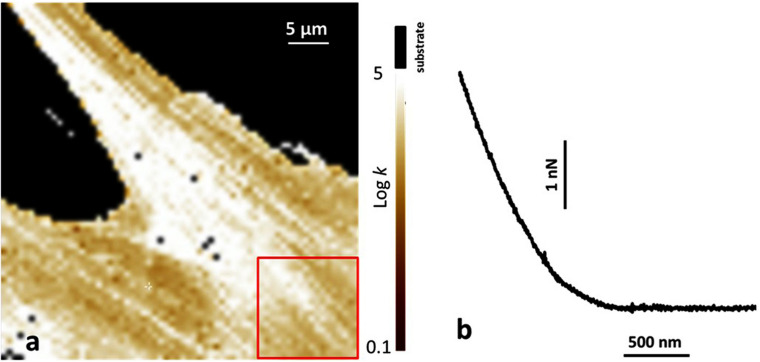
Atomic force microscopy (AFM) analysis of human bone marrow-derived mesenchymal stem cells (hBMSCs) grown on a plastic Petri dish and on Coll/Pro Osteon^®^ 200 biomaterial. **(a)** Map of the Young modulus of hBMSCs grown on a plastic Petri vessel. The value of the Young modulus is reported with a Log scale. The substrate is identified by the black region, as also reported on top of the color scale. The red square shows a typical area over which the Young modulus was averaged. **(b)** Typical example of a force curve obtained on cells on the biomaterial. The horizontal axis reports the tips-sample displacement, and the vertical axis reports the applied force.

**FIGURE 4:**
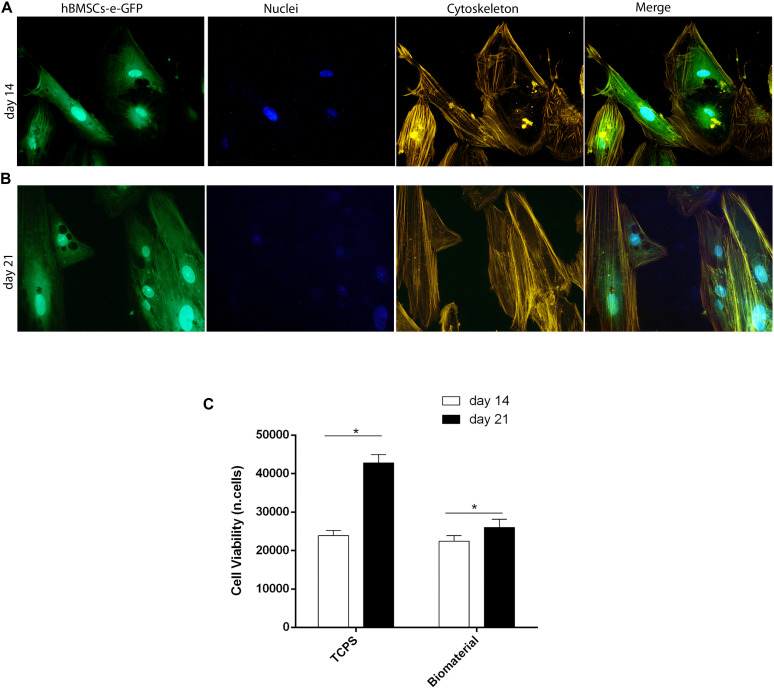
Stem cell viability, cytoskeleton architecture, and metabolic activity of human bone marrow-derived mesenchymal stem cells (hBMSCs) grown on scaffold. **(A)** Cytoskeleton architecture of recombinant hBMSC-enhanced green fluorescent protein (eGFP) cultures grown on the biomaterial, at day 14 (T1). **(B)** In this panel, actin filaments of hBMSC-eGFP grown on the biomaterial at day 21 do not show alteration in the structural organization compared to day 14, suggesting a good compatibility of the scaffold (magnification 20×). **(C)** hBMSC metabolic activity measured by AlamarBlue assay at days 14 and 21 of cultivation on the biomaterial. Statistically significant differences are evident between hBMSCs grown on biomaterials at days 14 and 21 (*p < 0.05). Tissue culture polystyrene (TCPS) exhibited the highest value in cell viability at day 21.

The scaffold showed its biocompatibility up to day 21. Indeed, cell adhesion, viability, and cytoskeleton organization of hBMSCs grown on the biomaterial were analyzed at days 14 and 21 (Figures 3, 4).

### Atomic Force Microscopy Analysis of Human Bone Marrow-Derived Mesenchymal Stem Cells Grown on Coll/Pro Osteon^®^ 200

Atomic force microscopy was used to investigate stem cells grown on both the biomaterial and TCPS. The Young modulus was studied and was compared between hBMSCs cells grown on petri dishes and cells grown on the biomaterial. We followed data obtained from the force curves exploiting the Sneddon model for the indentation process (Radmacher, 2007). In agreement with this model, the force F applied by the tip on the sample and the corresponding indentation δ is linked by the following equation: F = 2/πk/((1–υ^2^)) tagαδ^2^ in which (i) k is the sample Young modulus; (ii) α is the semi-angle of the cone representing the tip; and (iii) υ is the Poisson ratio of the cells, approximated to the value 0.5, characteristic of incompressible bodies. Figure 3a shows a typical example of the Young modulus map when the cells grow on the petri dishes. The representative value of the cell Young modulus was obtained considering the average value from selected regions of the cell, such as the one reported by the red square in Figure 3a. Instead, for the cells grown in contact with the biomaterial, due to its rough surface, it was not possible to obtain complete images of force volume, and the focus was on single force curves. In this case, all the values of the Young model obtained were averaged. Figure 3b reports a typical example of a force curve on cells growth on the biomaterial. Analysis showed two values similar to each other, such us values of (3.5 ± 2.0) kPa and (2.9 ± 1.7) kPa for the Young modulus of cells on the plastic petri dish and on the biomaterial, respectively.

### Human Bone Marrow-Derived Mesenchymal Stem Cell Viability

Cell viability was investigated through the direct morphology analysis of hBMSC-e-GFP cells grown on the biomaterial employing light and fluorescence microscopy (Figures 4A,B). Changes of phenotypic characteristics were evident in hBMSC-e-GFP grown on scaffold at days 14 (Figure 4A) and 21 (Figure 4B). It is important to remember that recombinant adenovirus allows *e-GFP* expression for only 3 weeks, which is needed to carry out the *in vitro* experiments.

### Cytoskeleton Analysis of Human Bone Marrow-Derived Mesenchymal Stem Cells Grown on the Scaffold

In order to analyze the cytoskeleton, hBMSC cultures expressing eGFP and grown on the scaffold were treated with phalloidin-TRITC staining at days 14 and 21 (Supplementary Figure 1). Results showed a well-organized cytoskeleton architecture, which has remained uninfluenced by the presence of the biomaterial up to day 21. In fact, at days 14 and 21 (Figures 4A,B), the actin filaments appeared unaltered, ascertaining the biocompatibility of tested scaffolds.

### Metabolic Activity of Human Bone Marrow-Derived Mesenchymal Stem Cells Grown on the Scaffold

Metabolic activity analyzed with AlamarBlue^TM^ assay increased in hBMSCs grown on the scaffold. The scaffold had a significant overall effect on viability at day 21 compared to day 14 (Figure 4C; *p* < 0.05). Based on the viability assay, the biomaterial did not elicit any cytotoxic effects; on the contrary, it induced cellular growth kinetics, which are statistically significant (*p* < 0.05).

### Osteogenic Markers

**FIGURE 5:**
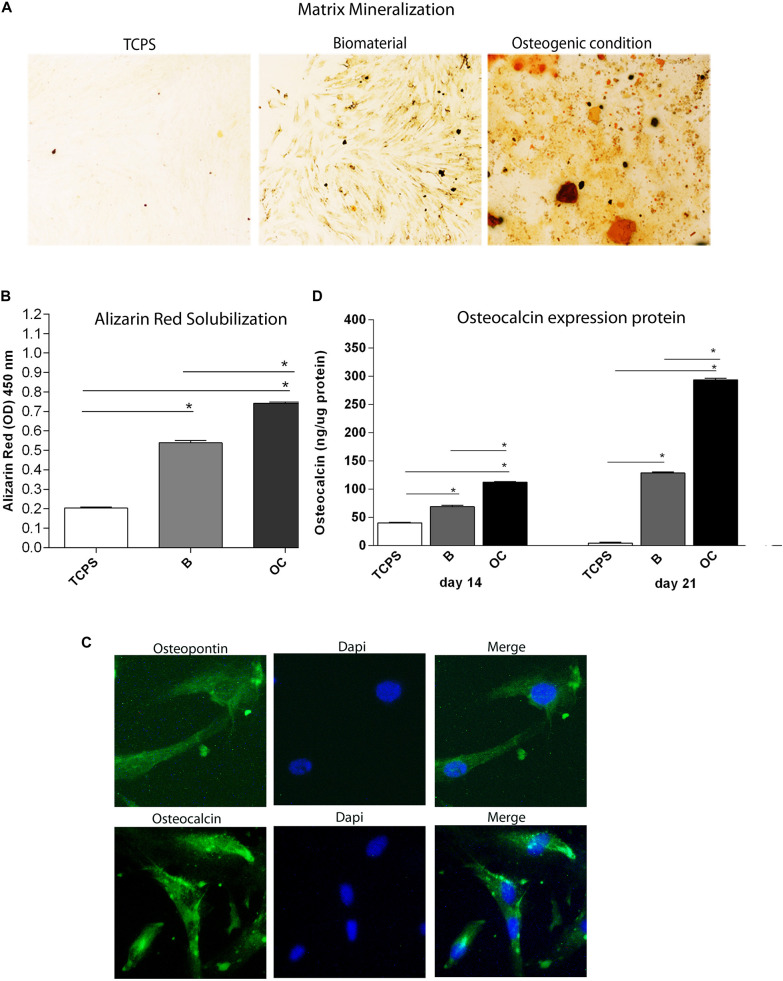
Osteogenic markers in human bone marrow-derived mesenchymal stem cells (hBMSCs) cultured on the biomaterial. **(A)** Alizarin red staining at day 21 is shown in the panel, in experimental conditions tested. Magnification 4×. **(B)** To quantify the amount of alizarin red staining in hBMSCs grown on the biomaterial, cells were eluted with 20% methanol and 10% acetic acid in a water solution and measured spectrophotometrically at 450 nm. Matrix mineralization data are reported as optical density and shown in the graph. **(C)** Detection of osteopontin and osteocalcin proteins by immunofluorescence staining in hBMSCs, at day 21. Symbols indicate statistical significance (*p < 0.001). Magnification 20×. **(D)** The temporal pattern of osteocalcin protein levels detected at different time points, i.e., at days 14 and 21, was quantified by ELISA. Osteocalcin protein was reported as ng of osteocalcin/1 μg of total protein.

In this study, the scaffold osteoinductive property is highlighted by matrix mineralization detected in hBMSCs grown on the scaffold, at day 21. To this purpose, cells were maintained in growth medium, without osteogenic supplements, such as dexamethasone, *b*-glycerophosphate, and ascorbic acid. To analyze late osteogenic differentiation, calcium mineral deposition and OPN and OCN proteins were analyzed at day 21 (Figure 5).

### Alizarin Red Human Bone Marrow-Derived Mesenchymal Stem Cell Staining

Alizarin red staining was performed to highlight the presence of mineralized (calcified) matrix areas in hBMSC cultures. hBMSCs grown on scaffold were stained with AR and imaged with bright-field microscopy. The biomaterial induces mineral matrix deposition better than the plastic vessel (TCPS), the control (Figures 5A,B). The quantification of AR was performed by eluting AR staining and acquiring optical density measurements. Osteogenic differentiation of hBMSCs grown on the biomaterial was increased three-fold than TCPS (*p* < 0.0001; Figure 5B). It should be noted that in OCs, the deposition of inorganic calcium salts was the most evident. In OC, the calcium deposits were higher than in cells grown on the scaffold and in TCPS (^∗∗^*p* < 0.0001) (Figure 5B).

### Osteopontin and Osteocalcin Expression Proteins on Human Bone Marrow-Derived Mesenchymal Stem Cell Cultures Grown on the Scaffold

After 21 days of culture, cells grown on the scaffold were stained with antibodies against OCN and OPN proteins to evaluate the effect of the biomaterial on osteogenic differentiation in hBMSCs. Images were visualized by fluorescence microscopy. Immunofluorescence staining revealed OCN- and OPN-positive hBMSC cultures grown on the scaffold at day 21 (Figures 5B,C), while the expression of these two osteogenic proteins was not detected in the TCPS (data not shown). In order to better understand differences in osteogenic markers in hBMSCs grown on the scaffold, OCN protein was quantified by ELISA hBMSCs actively expressed in the osteocalcin at day 14 (Figure 5D). Indeed, our data show a statistically significant increase in OCN levels in hBMSCs cultured on the biomaterial compared with TCPS at days 14 and 21 (^∗^*p* < 0.0001) (Figure 5D). Cells grown in OC had a higher level of OCN protein than hBMSCs grown on the biomaterial and in the control group (TCPS), at days 14 and 21 (^∗^*p* < 0.0001) (Figure 5D).

### Human Bone Marrow-Derived Mesenchymal Stem Cells Differentially Expressed Genes Implicated in Skeletal Development Are Modulated by Coll/Pro Osteon^®^ 200

**FIGURE 6:**
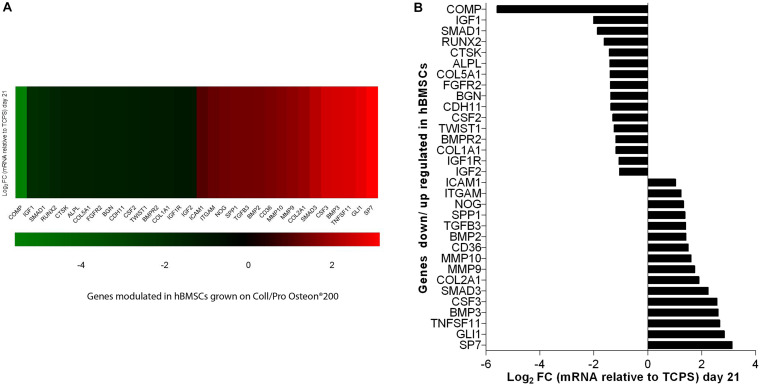
PCR array analyses of osteogenic genes expression in human bone marrow-derived mesenchymal stem cells (hBMSCs) grown on Coll/Pro Osteon^®^ 200 compared with tissue culture polystyrene (TCPS) reported at day 21. **(A)** Graphical representation through heat map of the mRNA expression in hBMSCs. The fold-change values (Log_2_ < 1 or > 1) is represented as up-regulated (red) and down-regulated (green) genes in hBMSCs grown on Coll/Pro Osteon^®^ 200 compared with the TCPS. **(B)** Graphical representation through bars of the mRNA expression in hBMSCs grown on Coll/Pro Osteon^®^ 200 compared with TCPS. Over-expressed genes (*n* = 16), at day 21, were transcription factor Sp7 (SP7); GLI family zinc finger 1 (GLI); TNF superfamily member 11 (TNFSF11); bone morphogenetic proteins 2 and 3 (BMP2 and BMP2 3); colony-stimulating factor 3 (CSF3); SMAD family member 3 (SMAD3); Collagen type II alpha 1 (*COL2A1*); matrix metallopeptidases 9 and 10 (MMP9 and MMP10); CD36 molecule (CD36); transforming growth factor, beta 3 (TGFB3)/RANKL; secreted phosphoprotein 1 (SPP1), Noggin (NOG); integrin, alpha M (ITGAM); intercellular adhesion molecule 1 (ICAM1). Down-regulated gene (*n* = 16) were cartilage oligomeric matrix protein (COMP); insulin growth factors 1 and 2 (IGF1, IGF2); SMAD family member 1 (SMAD1); runt-related transcription factor 2 (RUNX2); cathepsin K (CTSK), alkaline phosphatase (ALP); collagen type IV alpha 1 (COL5A1); fibroblast growth factor receptor 2 (FGFR2); biglycan (BGN); cadherin 11, type 2 (CDH11); colony-stimulating factor 2 (CSF2); twist family BHLH transcription factor 1 (TWIST1); bone morphogenetic protein receptor type II (BMPR2); Collagen type I alpha 1 (COL1A1); insulin growth factor 1 receptor (IGF1R); insulin growth factor 2 (IGF2).

In this investigation, the RT^2^ Profiler PCR array was used to analyze the expression levels of osteogenic genes in hBMSCs grown on Coll/Pro Osteon^®^ 200 compared with TCPS. Differentially expressed genes (DEGs; *n* = 32) including 16 up-regulated genes (Log_2_ FC > 1) and 16 down-regulated genes (Log_2_ FC < 1) were identified in hBMSCs grown on the biomaterial at day 21 (Table 1 and Figures 6A,B). At day 21, DEGs, which include the bone morphogenetic protein (BMP) 2/3 (BMP2/3), tumor necrosis factor ligand superfamily member 11 (TNFSF11/RANKL), matrix metallopeptidase 9/10 (MMP9/10), transforming growth factor (TGFβ3), secreted phosphoprotein 1 (SPP1), beta 3 (TGFβ3), and Noggin (NOG), which play important roles in ossification, were found to be up-regulated (Table 1 and Figure 6). Gene transcription factor Sp7 (SP7/Osterix) and SMAD family member 3 (SMAD3), which are two osteogenic transcription factors, were up-regulated in hBMSCs grown on the scaffold.

**TABLE 1:** Genes found to be up- or down-regulated in human bone marrow-derived mesenchymal stem cells (hBMSCs) grown on the scaffold at day 21.

Up-regulated genes			Down-regulated genes		
Number	Symbol/acronym	Fold-change (Log_2_ FC)	Number	Symbol/acronym	Fold-change (Log_2_ FC)
1	*SP7*	+3.13	1	*COMP*	−5.59
2	*GLI1*	+2.84	2	*IGF1*	−2.01
3	*TNFS11*	+2.67	3	*SMAD1*	−1.87
4	*BMP3*	+2.61	4	*RUNX2*	−1.62
5	*CSF3*	+2.57	5	*CTSK*	−1.43
6	*SMAD3*	+2.24	6	*ALPL*	−1.41
7	*COL2A1*	+1.90	7	*COL5A1*	−1.40
8	*MMP9*	+1.74	8	*FGFR2*	−1.39
9	*MMP10*	+1.61	9	*BGN*	−1.38
10	*CD36*	+1.50	10	*CDH11*	−1.37
11	*BMP2*	+1.42	11	*CSF2*	−1.30
12	*TGFB3*	+1.4	12	*TWST1*	−1.25
13	*SPP1*	+1.38	13	*BMPR2*	−1.19
14	*NOG*	+1.33	14	*COL1A1*	−1.19
15	*ITGAM*	+1.24	15	*IGF1R*	−1.07
16	*ICAM1*	+1.03	16	*IGF2*	−1.05

*Alkaline phosphatase (ALP); biglycan (BGN); bone morphogenetic proteins 2 and 3 (BMP2 and BMP3); bone morphogenetic protein receptor type II (BMPR2); cathepsin K (CTSK); cadherin 11, type 2 (CDH11); CD36 molecule (CD36); collagen type I alpha 1 (COL1A1); collagen type II alpha 1 (*COL2A1*); collagen type IV alpha 1 (COL5A1); colony-stimulating factor 2/3 (CSF2/3); cartilage oligomeric matrix protein (COMP); fibroblast growth factor receptor 2 (FGFR2); GLI family zinc finger 1 (GLI); intercellular adhesion molecule 1 (ICAM1); insulin growth factors 1 and 2 (IGF1, IGF2) and its receptor (IGF1R); insulin growth factor 2 (IGF2); integrin, alpha M (ITGAM); matrix metallopeptidases 9 and 10 (MMP9 and MMP10); Noggin (NOG); runt-related transcription factor 2 (RUNX2); SMAD family members 1 and 3 (SMAD1 and SMAD3); transcription factor Sp7 (SP7), secreted phosphoprotein 1 (SPP1), transforming growth factor, beta 3 (TGFB3)/RANKL; TNF superfamily member 11 (TNFSF11); twist family BHLH transcription factor 1 (TWIST1).*

Human BMSC cultures grown on the scaffold seem to be induced to chondrogenic differentiation from TCPS, as shown by *COL2A1* gene expression. DEGs include several growth factors, which were found to be up-regulated at day 21. Among these DEGs, there are colony-stimulating factor 3 (granulocyte-macrophage) (CSF3) and GLI1. In addition, genes encoding for cell ECM, adhesion molecules, such as CD36 molecule thrombospondin receptor (CD36), intercellular adhesion molecule 1 (ICAM1), integrin, and alpha M (ITGAM) were up-regulated, too. DEGs include those encoding for ECM molecules, such as Col type IV alpha 1 (COL5A1), Col type I alpha 1 (COL1A1), biglycan (BGN), and cathepsin K (CTSK), implicated in osteoclast, were down-regulated. Additionally, the insulin growth factors, such as growth factors 1/2 (IGF1/2), CSF 2 (CSF2), and IGF2, were tested to be down-regulated. Among the genes down-regulated, there were genes encoding for cell–cell adhesion molecules, such as cartilage oligomeric matrix protein (COMP) and cadherin 11 type 2 (CDH11). Early transcription factors, such as runt-related transcription factor 2 (RUNX2) and Twist1, were down-regulated (Table 1 and Figure 6). Up-regulated and down-regulated genes are reported (Table 1 and Figures 6A,B).

## Discussion

In this study, we employed hBMSC cultures to evaluate the biocompatibility and osteoinductive proprieties of a bone substitute, which is highly close to the natural bone structure made of HA (Pro Osteon^®^ 200) dispersed in Col. Bone regrowth, as well as regenerative medicine in general, has emerged as a multidisciplinary field, which takes advantage of knowledge in different sciences, such as materials science, cell biology, tissue engineering, molecular genetics, and epigenetics. All these fields contribute to repairing or regenerating damaged tissues through the atypical combination of three-dimensional (3D) biomaterial scaffolds, signaling molecules, and stem cells (Iaquinta et al., 2019a,b; Sheehy et al., 2019). In order to identify the best/right MSCs to be employed in therapies and for the correct characterization of the scaffold to be used, it is necessary to analyze different types of human MSCs using *in vitro* approaches. Comparative data regarding hBMSC cultures for adhesion on biomaterials and related biocompatibility to various biomaterials are lacking to a large extent. A number of growth factors, cytokines, drugs, gene products, and mechanical scaffold proprieties are critical for differentiating stem cells, including osteoblasts. The precise expression pattern depends on a balance of positive and negative transcription factors, proteins that control mRNA synthesis from the specific gene (Okazaki and Sandell, 2004; Tang et al., 2019). In this investigation, SEM images confirmed that incorporating Col fibers in HA/Pro Osteon^®^ 200 make a biomimetic porous structure composed of HA and Col. Our data demonstrate that the HA/derived scaffold provides a good microenvironment for hBMSC adhesion and viability.

Stem cells grown on the biomaterial and those in contact with plastic vessels were analyzed using the AFM. Indeed, AFM because of its ability to operate in almost physiological conditions has been largely exploited in last decades to study the nanomechanical properties and mechano-response of cells to the external microenvironment (Kasas et al., 2018; Krieg et al., 2019). Cell stiffness, generally quantified in terms of the Young modulus, represents the main parameters extracted from AFM measurements on cells. Eventual changes of these features entail relevant variation in cytoskeleton structure reorganization, including actin fibers and focal adhesion complexes with matrix. The Young modulus of hBMSCs cells on petri dishes was compared with the same parameter for cells grown on the biomaterial. From the analysis, we obtained the values of (3.5 ± 2.0) kPa and (2.9 ± 1.7) kPa for the Young modulus of cells on the plastic petri dish and on the biomaterial, respectively. AFM measurements showed no significant differences of the Young modulus for cells grown on the biomaterial and in contact with plastic petri dish. This result corroborates the fact that cytoskeleton organization is similar in both samples and is not influenced by the scaffold under analysis.

Cell morphology of eGFP-hBMSC cells was indistinguishable from parental hBMSCs. Cytoskeleton architecture appeared well-organized, whereas its integrity remained uninfluenced by the biomaterial. Metabolic activity was increased during experiments on hBMSCs grown on the biomaterial. Previous studies reported that a pivotal role for hASC osteogenic differentiation is played by the HA-derived scaffold Coll/Pro Osteon^®^ 200, whereas HA and Col mainly support survival and viability (Mazzoni et al., 2017, 2020).

In this investigation, the osteoinductive activity of the biomaterial is highlighted by the matrix mineralization detected in hBMSCs grown on the scaffold, at day 21. Immunohistochemistry reveals the expression of the two osteogenic markers, such as the OPN and OCN proteins in hBMSCs grown on Coll/Pro Osteon^®^ 200 at day 21. ELISA data show a statistically significant increase in osteocalcin protein expression in hBMSCs grown on the biomaterial compared with the control at days 14 and 21. This result is in agreement with previous data, where the hybrid scaffold was able to influence hASC osteogenic pathway with an up-regulation of OCN protein compared with TCPS (Mazzoni et al., 2020).

Expression of 84 osteogenic genes was evaluated by quantitative PCR (Q-PCR) array technologies. In hBMSCs, the scaffold induces up-regulation of specific genes that are involved in the ossification process, such as *SP7*, *SMAD3*, *BMP2/3*, *TGF-β3*, *NOG*, and *SPP1*. Genes that codify for osteogenic transcription factors *SP7* and *SMAD3* were up-regulated in hBMSCs grown on the scaffold.

Osteogenesis is the process of new bone formation where transcription factors play an important role in controlling cell proliferation and differentiation (Gomathi et al., 2020). It is reported that *Sp7* is a transcription factor for osteoblast differentiation, whereas *Runx2* is known as a downstream gene (Xin et al., 2019). *BMP2* pathway is known to up-regulate *Sp7* expression through two distinct transcription factors such as *Runx2* and *Msx2* during osteoblast differentiation (Li et al., 2019; Tang et al., 2019). Accumulating data proved that BMP2 is involved in bone formation, bone remodeling, bone development, and osteoblast differentiation through hBMSC osteogenic differentiation (Biswas et al., 2018; Wu et al., 2018). BMPs are pleiotropic ligands in the TGF-β superfamily, which contains TGF-β/SMAD3. In agreement with our observation, BMP2 was shown to activate SMAD3-dependent signaling (Ongaro et al., 2019). Transcription factor *SMAD3* expression is crucial for osteogenesis and the skeletal development process (Lin et al., 2019). Some studies have demonstrated that TGF-β3 also recruits endogenous human MSCs to initiate bone regeneration. *TGF-β3* induces endochondral bone formation and completes bone remodeling. The signal transduction mediated by *TGF-β 3* in osteogenic differentiation and bone regeneration specifically occurs through both canonical *SMAD*-dependent pathways with *TGF-β3* ligands, receptors, and SMADs (Li et al., 2019). Indeed, *TGFβ3* gene and *SMAD3* transcription factor up-regulation could induce bone regeneration by amplifying hBMSCs recruitment at the damaged site (Deng et al., 2017).

In a previous study, other stem cells, such as hASCs, grown on the scaffold Coll/Pro Osteon^®^ 200, did not show up-regulation of *SMAD* gene expression (Mazzoni et al., 2020). Osteoblastic differentiation of hASCs induced by Coll/Pro Osteon^®^ 200 at day 21 appears to occur without the up-regulation of *SMAD* genes; otherwise, *SMAD3* genes are up-regulated in hBMSCs grown on the HA/hybrid scaffold at day 21. In agreement with a previous study (Mazzoni et al., 2020), the *SPP1* gene was found to be expressed with a fold change (Log_2_ FC) of 1.38. *SPP1* codes for one of the most predominant non-collagenous proteins in bone ECM produced by osteoblasts, which also promotes cell adhesion to the bone surface (Sodek et al., 2000). We detected up-regulation of the *COL2A1* gene expression in hBMSCs grown on HA-derived scaffold with a fold change (Log_2_ FC) of 1.90. Col, type II, alpha 1, encoded by the gene *COL2A1*, is the major Col in articular cartilage synthesized by chondrocytes (Rolvien et al., 2020). Herein, up-regulation of *TNFS11*/*RANKL* was detected. RT^2^ Profiler PCR arrays revealed an increased expression of RANKL with a fold change (Log_2_ FC) of 2.67. Receptor activator of RANKL, a member of the TNF superfamily, mainly controls later phases of osteoclast differentiation (Hodge et al., 2007; Hiyama et al., 2019). The bone resorption function of osteoclasts in the development of the skeleton and in mineral homeostasis requires a balance in bone-forming osteoblast activities. Indeed, a pivotal factor in the health and maintenance of bone density is the coordinated activity of osteoblasts and osteoclasts. In our experiments, *MMP9* and *MMP10* expression was up-regulated at day 21. ECM molecules, such as *MMP9* and *MMP10*, play a central role in the development of skeletal tissues, orthopedic diseases and trauma, such as fracture/osteotomy repair, and congenital skeletal deformity. MMPs play an active role in the formation of osteoid tissue, which is rich in collagens and other ECM proteoglycans (Paiva and Granjeiro, 2017; Mianehsaz et al., 2019). A recent study has reported that the deletion of the matrix metalloproteinase MMP9 inhibited human osteoclast activity, increased bone density, and prevented pathogenic bone loss (Zhu et al., 2020). MMP10 takes part in physiological processes, like bone growth (Ortega et al., 2004). Other authors have shown that MMP10 enhances BMP2-induced osteoblast differentiation *in vitro*. These data were confirmed by another study that showed a similar range of dose and that proportions of *BMP2* and *MMP10* are required *in vivo* for their synergistic osteogenic effect to induce bone regeneration (Reyes et al., 2018). *ITGAM* gene expression was up-regulated in hBMSCs grown on the scaffold. Tissue repair and regeneration are highly complex, whereas dynamic processes involve the coordinated efforts of many different cellular, humoral, and molecular pathways including integrins (Paiva and Granjeiro, 2017). Endothelial NOG expression in bone promoted by activating Notch and hypoxia-inducible factor 1 alpha (HIF-1a) signals could induce proliferation and differentiation of perivascular osteoprogenitors and then promote osteogenesis (Ramasamy et al., 2014).

In hBMSCs grown on the scaffold, down-regulated genes encoding for ECM and cell-to cell adhesion molecules, such as *BGN*, *CDH11*, *COL1A1*, *COL5A1*, *COMP*, *CSF2*, *CTSK*, *IGF1/2*, *IGF1R*, and *ALPL*, were identified. Early transcription factors, such as *RUNX2*, *SMAD1*, and *TWIST1*, were also down-regulated at day 21. Protein *Runx2*, a key transcription factor, regulates the differentiation of MSCs into osteoblasts, which further mature into osteocytes. We may speculate that *Runx2* down-regulation may contribute to the osteoblast maturation effect. *Runx2* expression may have been up-regulated earlier than day 21.

It would be possible that the ossification, induced herein by scaffold/hBMSCs, may have taken place at earlier time compared with scaffold/hASCs reported in a previous report (Mazzoni et al., 2020). Indeed, in previous studies, data obtained in hASCs showed that the *Runx2* transcription factor expression gene was up-regulated up to day 21 (Mazzoni et al., 2020). Our results show that in hBMSCs, several osteogenic genes are up-modulated such as BMP2/3, SPP1, and transcription factors such as SMAD3 and SP7 when the stem cells grown on the scaffold are composed of HA (Pro Osteon^®^ 200) and Collagen (Avitene^TM^ Microfibrillar Collagen Hemostat) for 21 days. In addition, hBMSCs had a positive epigenetic modulation on genes for cartilage condensation (*COL2A1*) and osteoclast maturation (TNSF11/RANKL) at day 21. Array approach revealed that 16 genes are up-regulated in hBMSCs grown on the scaffold, at day 21, while in previous studies, we quantified 22 genes involved in bone development as up-regulated in hASC cultures grown on the same Coll/Pro Osteon^®^ 200 scaffold. hASC cultures grown on the scaffold seem to maintain a positive modulation of osteogenic genes as compared with hBMSCs, up to day 21, without showing any negative modulation of osteogenic genes.

## Conclusion

Our experiments suggest that the innovative scaffold composed of Granular Pro Osteon^®^ 200 coralline HA and Avitene^TM^ Microfibrillar Collagen Hemostat, tested herein, provides a good microenvironment for hBMSC adhesion, viability, and osteoinduction. In addition, our morphology, cell biology, and epigenetic analyses suggest that hBMSCs represent an excellent *in vitro* cellular model to test scaffolds to be used for bone repair/regrowth and tissue engineering.

## Data Availability Statement

The raw data supporting the conclusions of this article will be made available by the authors, without undue reservation.

## Ethics Statement

A bone marrow biopsy was collected after the written informed consent obtained from patients of the Orthopedic Institute “Rizzoli” Bologna, Italy, under protocol approved by the Ethical Committee of Bologna, Italy.

## Author Contributions

EM, AD’A, LT, RN, FM, and MT contributed to the conception of the study. FF performed the fluorescence-activated cell sorting (FACS) analysis. CM, MI, and CL performed cellular and molecular experiments. GB-B provided patients’ samples. AM and AA performed AFM experiments. EM, FM, and MT wrote the original draft. All authors contributed to the article and approved the submitted version.

## Conflict of Interest

The authors declare that the research was conducted in the absence of any commercial or financial relationships that could be construed as a potential conflict of interest.

## Supplementary Material

Supplementary Figure 1 Stem cell viability and cytoskeleton architecture assays carried out in hBMC monolayers grown in 24 well tissue culture polystyrene (TCPS). Representative images of hBMC cultures obtained by fluorescent microscope analysis. **(A)** hBMCs expressing green fluorescence; **(B)** HBMC cytoskeleton analysis by phalloidin TRITC (tetramethylrhodamine isothiocyanate) staining; **(C)** Cellular nuclei stained with 0.5 mg/ml DAPI; **(D)** Cytoskeleton analysis of hBMCs, expressing eGFP, by phalloidin TRITC staining.
