# Supplementary figures and images for: Enhanced Osteogenic Differentiation of Human Bone Marrow-Derived Mesenchymal Stem Cells by a Hybrid Hydroxylapatite/Collagen Scaffold
Source: Front Cell Dev Biol. 2021 Jan 11;8:610570. doi: 10.3389/fcell.2020.610570 (PMC7849836; doi:10.3389/fcell.2020.610570)

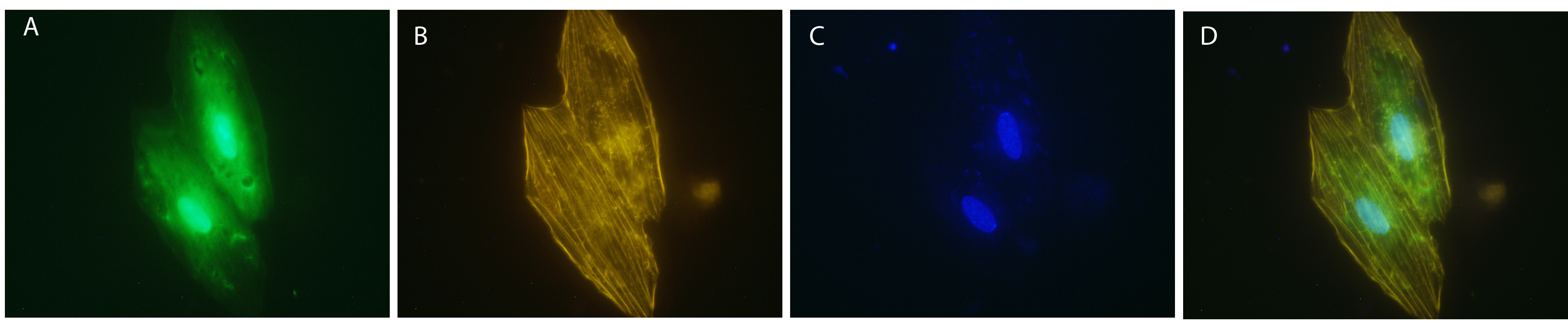

Supplement: Supplementary Figure 1 — Stem cell viability and cytoskeleton architecture assays carried out in hBMC monolayers grown in 24 well tissue culture polystyrene (TCPS). Representative images of hBMC cultures obtained by fluorescent microscope analysis. (A) hBMCs expressing green fluorescence; (B) HBMC cytoskeleton analysis by phalloidin TRITC (tetramethylrhodamine isothiocyanate) staining; (C) Cellular nuclei stained with 0.5 mg/ml DAPI; (D) Cytoskeleton analysis of hBMCs, expressing eGFP, by phalloidin TRITC staining. [file Image_1.TIF]
